# Risk of Inflammatory Bowel Disease Following Appendectomy in Adulthood

**DOI:** 10.3389/fmed.2021.661752

**Published:** 2021-06-02

**Authors:** Wei-Sheng Chung, Sunny Chung, Chung-Y Hsu, Cheng-Li Lin

**Affiliations:** ^1^Department of Internal Medicine, Taichung Hospital, Ministry of Health and Welfare, Taichung, Taiwan; ^2^Department of Health Services Administration, China Medical University, Taichung, Taiwan; ^3^Department of Healthcare Administration, Central Taiwan University of Science and Technology, Taichung, Taiwan; ^4^Department of Chemistry, Point Loma Nazarene University, San Diego, CA, United States; ^5^Graduate Institute of Biomedical Sciences, China Medical University, Taichung, Taiwan; ^6^Management Office for Health Data, China Medical University Hospital, Taichung, Taiwan; ^7^College of Medicine, China Medical University, Taichung, Taiwan

**Keywords:** appendectomy, inflammatory bowel disease, ulcerative colitis, Crohn's disease, cohort study

## Abstract

**Background:** The appendix has a complicated immune function, and appendectomy may derange the immune system. Studies on the relationship between appendectomy and subsequent inflammatory bowel disease (IBD) have been inconsistent. We conducted a nationwide cohort study consisting of individuals who underwent appendectomy to evaluate the incidence and risk of ulcerative colitis (UC) and Crohn's disease (CD).

**Methods:** We identified patients aged >20 years who underwent appendectomy between 2000 and 2012 from inpatient claims of the National Health Insurance Research Database (NHIRD) and assigned them to the appendectomy cohort. Then, we randomly selected patients without appendectomy in the NHIRD and assigned them to the comparison cohort in a frequency-matched 1:1 ratio based on sex, age, and index year. We tracked down all participants until IBD diagnosis, death, or the end of 2013. Cox models were used to estimate the hazard ratio (HR), and 95% confidence intervals (CIs) were used to compare the IBD risk between the appendectomy and comparison cohorts.

**Results:** The appendectomy and comparison cohorts in the study consisted of 246 562 patients each. The appendectomy cohort exhibited a 2.23- and 3.48-fold higher risk of UC (adjusted HR = 2.23, 95% CI = 1.59-3.12) and CD (adjusted HR = 3.48, 95% CI = 2.42-4.99), respectively, than did the comparison cohort. UC and CD risks significantly increased in the appendectomy cohort regardless of whether appendicitis was present.

**Conclusions:** Our study suggests that appendectomy increases UC and CD risks irrespective of appendicitis.

## Introduction

The human appendix is similar to a diverticulum of the cecum, which is considered a vestigial organ. Previously, the biological function of the appendix was unclear; therefore, it was surgically removed on inflammation. Clinicians consider appendectomy a safe and effective technique for managing appendicitis (1). In the United States, the lifetime appendectomy risks in men and women are 12 and 23% but the lifetime appendicitis risks are 8.6 and 6.7%, respectively (2). In Taiwan, 10.8% of appendectomies were not related to appendicitis (3).

The submucosa of the appendix contains numerous lymphoid follicular centers. In addition, complicated immune system cells are present in the mucosa (e.g., Treg cells, M cells, and T and B cells) and submucosa (e.g., B lymphocytes, T lymphocytes, macrophages, centrocytes, and CD4^+^/CD8^+^ cells) of the appendix (4, 5). Studies have indicated that the appendix interacts with intestinal flora and balances the intestinal immune system (4–7). The bacteria in the appendix may act as a biofilm inoculum of the intestinal commensal microbiome, which facilitates reinoculation of the proximal large bowel and terminal ileum. The complex immune system and a shelter for microbiome in the appendix can balance proinflammation and antiinflammation of the bowel and maintain homeostasis (4).

Inflammatory bowel diseases (IBDs) mainly consist of Crohn's disease (CD) and ulcerative colitis (UC), which cause prolonged inflammation of the digestive tract. IBDs considerably affect health-related quality of life and markedly increase health care costs (8, 9). The incidence of IBDs has increased steadily in Taiwan (10). The exact cause of IBDs remains unclear. In addition to genetics, the environmental composition of intestinal microbiome may play a role in uncontrolled gut inflammation (11). However, the relationship between appendectomy and IBD development has been controversial in Western countries (12–14). Studies on the association of appendectomy with subsequent IBD risk are scant. Therefore, we conducted a nationwide cohort study to examine whether IBD risk is higher in the appendectomy cohort than in the non-appendectomy cohort.

## Methods

### Data Source

The data for this study were obtained from the National Health Insurance Research Database (NHIRD). The Taiwan government launched a National Health Insurance Program (NHIP) in 1995. NHIP provides comprehensive medical care services to nearly all (99%) residents in Taiwan. The health information, which includes claims of the inpatients and outpatients and medications prescribed, was recorded in the NHIRD. For patient privacy, the data were deidentified. In this cohort study, we used all inpatient data from 2000 to 2013.

### Study Sample

The present cohort study consisted of case and comparison groups. People who underwent appendectomy (International Classification of Diseases, Ninth Revision, Clinical Modification [*ICD-9-CM*] codes 47.0 and 47.1) were recruited as the case group.

We defined the first operation date of appendectomy as the index date. Patients with IBD diagnosis (*ICD-9-CM* codes 555 and 556) before the index date were excluded. The comparison group consisted of people without a history of IBDs and appendectomy. Participants aged <20 years were excluded from both the groups. Controls were matched with patients in the case group based on sex, age, and index year with a 1:1 ratio. All participants were followed from the index date to withdrawal from the NHIP, death, or end of the study (December 31, 2013).

### Main Outcomes and Covariates

The endpoint of this study was defined as IBD diagnosis (*ICD-9-CM* codes 555 and 556), including UC (*ICD-9-CM* code 556) and CD (*ICD-9-CM* code 555). We divided participants into 3 age groups: 20–34 years, 35–49 years, and >50 years. Some IBD-related medical comorbidities, including hypertension (*ICD-9-CM* codes 401–405), diabetes (*ICD-9-CM* code 250), hyperlipidemia (*ICD-9-CM* code 272), cardiovascular disease (*ICD-9-CM* codes 430–438), heart failure (*ICD-9-CM* code 428), chronic obstructive pulmonary disease (*ICD-9-CM* codes 491, 492, and 496), chronic kidney disease (*ICD-9-CM* codes 580–589), alcohol-related diseases (*ICD-9-CM* codes 291, 303, 305.00, 305.01, 305.02, 305.03, 571.0–571.3, 790.3, and V11.3), cirrhosis (*ICD-9-CM* code 571), and biliary stones (*ICD-9-CM* code 574), were considered potential confounding factors.

### Statistical Analysis

A chi-square test was used for comparing the categorical variables between case and comparison groups. The mean age was examined using the student *t*-test. Then, we calculated the IBD incidence rates (per 10,000 person years) among the two groups. Hazard ratios (HRs) were estimated using a Cox proportional hazards model and adjusted using the variables of age, sex, and comorbidities into the multivariable Cox proportional hazards model. Furthermore, we analyzed the association between the follow-up period and IBDs. The cumulative incidences of IBDs in the 2 groups were estimated using the Kaplan–Meier method. Furthermore, the log-rank test was used to evaluate the difference between the two curves. The statistical significance level was represented by a *P*-value of <.05 for all tests.

## Results

A total of 493,124 participants were included in this study. The mean follow-up time for the appendectomy group was 7.05 (±3.91) years, and that for the comparison group was 7.17 (±3.88) years. The distribution of demographics and comorbidities between appendectomy patients and controls is presented in Table 1. The proportions of age groups and gender were not significantly different between the 2 groups. Most of the study population was in the age group of 20–34 years (38.5%) and most participants were male (51.4%). The mean age of the case group was 43.3 (±16.7) years and that of the control group was 43.1 (16.9) years. The proportion of patients with comorbidities was significantly higher in the appendectomy group than in the control group.

**Table 1:** Comparison of demographics and comorbidities between patients with and without appendectomy.

	**Appendectomy**		
	**Yes (*N* = 246,562)**	**No (*N* = 246,562)**	
	***n* (%)**	***n* (%)**	***p*-value**
Age, years			0.99
20–34	94,814 (38.5)	94,814 (38.5)	
35–49	74,812 (30.3)	74,812 (30.3)	
>50	76,936 (31.2)	76,936 (31.2)	
Mean (SD)^†^	43.3 (16.7)	43.1 (16.9)	<0.001
Gender			0.99
Female	119,806 (48.6)	119,806 (48.6)	
Male	126,756 (51.4)	126,756 (51.4)	
Comorbidity			
CAD	8,657 (3.51)	6,011 (2.44)	<0.001
Hypertension	26,628 (10.8)	13,128 (5.32)	<0.001
Diabetes	14,566 (5.91)	7,602 (3.08)	<0.001
Hyperlipidemia	6,080 (2.47)	3,815 (1.55)	<0.001
CVA	6,764 (2.74)	5,462 (2.22)	<0.001
Heart failure	2,837 (1.15)	1,845 (0.75)	<0.001
COPD	4,237 (1.72)	2,758 (1.12)	<0.001
CKD	2,056 (0.83)	834 (0.34)	<0.001
Alcohol-related diseases	2,250 (0.91)	1,282 (0.52)	<0.001
Cirrhosis	8,729 (3.54)	4,529 (1.84)	<0.001
Biliary stone	6,167 (2.50)	3,070 (1.25)	<0.001

*Categorical data were examined using a chi-squared test*;

† *continuous data were examined using a t-test*.

*CAD, coronary artery disease; CKD, chronic kidney disease; COPD, chronic obstructive pulmonary disease; CVA, cerebrovascular disease; SD, standard deviation*.

Table 2 shows the incidence and HRs of IBDs. The incidence rate of IBDs in the case group was 14.7 per 10,000 person years, and that in the comparison group was 4.92 per 10,000 person years. The IBD risk in patients with appendectomy was 2.78-fold higher than (95% confidence interval [CI] = 2.17–3.55) that in people without appendectomy. Appendectomy increases UC risk by 2.23 times (95% CI = 1.59–3.12). The adjusted HR of CD for appendectomy patients compared with controls was 3.48 (95% CI = 2.42–4.99). Appendectomy increased IBD risk regardless of gender or age groups. The adjusted HR of IBDs was higher in patients with appendectomy than in patients without appendectomy irrespective of comorbidities (adjusted HR = 3.12, 95% CI = 2.34–4.17 in patients without comorbidity and adjusted HR = 1.75, 95% CI = 1.11–2.77 in patients with comorbidity, respectively).

**Table 2:** Incidence and adjusted hazard ratio of inflammatory bowel disease based on sex, age, and comorbidities for patients with appendectomy compared with controls.

	**Appendectomy**						**Compared to control**	
	**Yes**			**No**				
**Variables**	**Events**	**PY**	**Rate^#^**	**Events**	**PY**	**Rate^#^**	**Crude HR (95% CI)**	**Adjusted HR^†^ (95% CI)**
All	255	1,737,942	14.7	87	1,766,861	4.92	2.98 (2.33, 3.79)1^***^	2.78 (2.17, 3.55)^***^
Ulcerative colitis (UC)	116		6.67	49		2.77	2.40 (1.72, 3.36)^***^	2.23 (1.59, 3.12)^***^
Crohn's disease (CD)	139		8.00	38		2.15	3.71 (2.59, 5.31)^***^	3.48 (2.42, 4.99)^***^
Gender								
Female	119	851,339	14.0	38	863,289	4.40	3.17 (2.20, 4.57)^***^	2.95 (2.04, 4.26)^***^
Male	136	886,602	15.3	49	903,572	5.42	2.82 (2.04, 3.91)^***^	2.64 (1.90, 3.67)^***^
Age, years								
≤34	101	713,152	14.2	29	704,429	4.12	3.45 (2.28, 5.21)^***^	3.33 (2.20, 5.04)^***^
35–49	64	553,971	11.6	13	560,303	2.32	4.98 (2.74, 9.03)^***^	4.63 (2.54, 8.44)^***^
>50	90	470,819	19.1	45	502,128	8.96	2.13 (1.49, 3.04)^***^	1.81 (1.26, 2.60)^**^
Comorbidity								
No	175	146,6719	11.9	63	1,628,953	3.87	3.09 (2.32, 4.12)^***^	3.12 (2.34, 4.17)^***^
Yes	80	271,222	29.5	24	137,908	17.4	1.69 (1.07, 2.67)^*^	1.75 (1.11, 2.77)^*^

*Rate^#^, incidence rate per 10,000 person-years; Crude HR, relative hazard ratio*;

*Adjusted HR^†^, adjusted hazard ratio after control for age, sex, and comorbidities of CAD, hypertension, diabetes, hyperlipidemia, CVA, heart failure, COPD, CKD, alcohol-related diseases, cirrhosis, and biliary stone*.

*CAD, coronary artery disease; CI, confidence interval; CKD, chronic kidney disease; COPD, chronic obstructive pulmonary disease; CVA, cerebrovascular disease; PY, person years; SD, standard deviation*.

* *P < 0.05*,

** *P < 0.01*,

*** *P < 0.001*.

The incident rates and HRs of IBDs stratified based on the follow-up period are presented in Table 3. The adjusted HRs of IBDs for patients with appendectomy relative to controls were 3.99 (95% CI = 2.69–5.91), 2.67 (95% CI = 1.64–4.35), and 1.74 (95% CI = 1.14–2.66) in people with a follow-up time of <3, 3–6, and >6 years, respectively. For UC and CD, the highest adjusted HR was observed in patients with a follow-up time of <3 years.

**Table 3:** Incidence and adjusted hazard ratio of inflammatory bowel disease based on follow-up period for patients with appendectomy compared with controls.

	**Appendectomy**						**Compared to control**	
	**Yes**			**No**				
**Variables**	**Events**	**PY**	**Rate^#^**	**Events**	**PY**	**Rate^#^**	**Crude HR (95% CI)**	**Adjusted HR^†^ (95% CI)**
All								
Follow-up time, years								
<3	131	678,513	19.3	31	685,753	4.52	4.26 (2.88, 6.31)^***^	3.99 (2.69, 5.91)^***^
3–6	64	504,262	12.7	22	512,187	4.30	2.96 (1.82, 4.80)^***^	2.67 (1.64, 4.35)^***^
>6	60	555,167	10.8	34	568,921	5.98	1.81 (1.19, 2.75)^**^	1.74 (1.14, 2.66)^*^
Ulcerative colitis (UC)								
Follow-up time, years								
<3	54		7.96	15		2.19	3.63 (2.05, 6.44)^***^	3.30 (1.85, 5.88)^***^
3–6	32		6.35	11		2.15	2.96 (1.49, 5.86)^**^	2.67 (1.34, 5.34)^**^
>6	30		5.40	23		4.04	1.34 (0.78, 2.30)	1.28 (0.74, 2.22)
Crohn's disease (CD)								
Follow-up time, years								
<3	77		11.4	16		2.33	4.85 (2.83, 8.32)^***^	4.58 (2.67, 7.87)^***^
3–6	32		6.35	11		2.15	2.95 (1.49, 5.86)^**^	2.65 (1.33, 5.29)^**^
>6	30		5.40	11		1.93	2.80 (1.40, 5.58)^**^	2.69 (1.34, 5.40)^**^

*Rate^#^, Incidence rate per 10,000 person-years; Crude HR, relative hazard ratio*;

*Adjusted HR^†^, adjusted hazard ratio after control for age, sex, and comorbidities of CAD, hypertension, diabetes, hyperlipidemia, CVA, heart failure, COPD, CKD, alcohol-related diseases, cirrhosis, and biliary stone*.

*CAD, coronary artery disease; CI, confidence interval; CKD, chronic kidney disease; COPD, chronic obstructive pulmonary disease; CVA, cerebrovascular disease; PY, person years; SD, standard deviation*.

* *P < 0.05*,

** *P < 0.01*,

*** *P < 0.001*.

Table 4 presents the incidence and risk of UC and CD for the appendectomy cohort without appendicitis and with appendicitis compared with those for the non-appendectomy cohort. The incidence and risk of UC (13.4 vs. 2.77 per 10 000 person years, adjusted HR = 3.19, 95% CI = 1.86–5.50) and CD (14.8 vs. 2.15 per 10,000 person years, adjusted HR = 6.13, 95% CI = 3.54–10.6) were substantially higher in the appendectomy cohort without appendicitis than in the non-appendectomy cohort. However, the incidence and risk of UC (6.08 vs. 2.77 per 10,000 person years, adjusted HR = 2.11, 95% CI = 1.49–2.98) and CD (7.39 vs. 2.15 per 10,000 person years, adjusted HR = 3.24, 95% CI = 2.24–4.68) were higher in the appendectomy cohort with appendicitis than in the non-appendectomy cohort.

**Table 4:** Comparison of the HRs of ulcerative colitis and Crohn's disease among patients who underwent appendectomy with and without appendicitis and controls.

**Variable**	**N**	**Event**	**Rate^#^**	**Crude HR (95% CI)**	**Adjusted HR^†^ (95% CI)**
Ulcerative colitis					
Non-appendectomy	246,562	49	2.77	1.00	1.00
Appendectomy					
Without appendicitis	21,996	19	13.4	4.81 (2.83, 8.16)^***^	3.19 (1.86, 5.50)^***^
With appendicitis	224,566	97	6.08	2.19 (1.55, 3.09)^***^	2.11 (1.49, 2.98)^***^
Crohn's disease					
Non- appendectomy	246,562	38	2.15	1.00	1.00
Appendectomy					
Without appendicitis	21,996	21	14.8	6.77 (3.97, 11.5)^***^	6.13 (3.54, 10.6)^***^
With appendicitis	224,566	118	7.39	3.43 (2.38, 4.95)^***^	3.24 (2.24, 4.68)^***^

*Rate^#^, incidence rate per 10,000 person-years; Crude HR, relative hazard ratio*;

*Adjusted HR^†^, adjusted hazard ratio after control for age, sex, and comorbidities of CAD, hypertension, diabetes, hyperlipidemia, CVA, heart failure, COPD, CKD, alcohol-related diseases, cirrhosis, and biliary stone*.

*CAD, coronary artery disease; CI, confidence interval; CKD, chronic kidney disease; COPD, chronic obstructive pulmonary disease; CVA, cerebral vascular disease; PY, person years; SD, standard deviation*.

*** *P < 0.001*.

Table 5 lists the incidence and risk of UC and CD among patients of various ages with appendectomy compared with the corresponding controls. The incidence and risk of UC and CD were significantly higher in the appendectomy cohort irrespective of when appendectomy was conducted than in the corresponding controls.

**Table 5:** Comparison of the HR for ulcerative colitis and Crohn's disease among patients who underwent appendectomy at different ages.

	**Appendectomy**						**Compared to control**	
	**No**			**Yes**				
**Variables**	**Events**	**PY**	**Rate^#^**	**Events**	**PY**	**Rate^#^**	**Crude HR (95% CI)**	**Adjusted HR^†^ (95% CI)**
Ulcerative colitis (UC)	49	176,6861	2.77	116	1,737,942	6.67	2.40 (1.72, 3.36)^***^	2.23 (1.59, 3.12)^***^
Age, years								
≤34	15	704,429	2.13	35	713,152	4.91	2.31 (1.26, 4.22)^***^	2.17 (1.18, 3.99)^***^
35–49	8	560,303	1.43	27	553,971	4.87	3.42 (1.55, 7.52)^***^	3.41 (1.54, 7.54)^**^
>50	26	502,128	5.18	54	470,819	11.5	2.21 (1.39, 3.53)^***^	1.89 (1.17, 3.04)^**^
Crohn's disease (CD)	38	176,6861	2.15	139	1,737,942	8.00	3.71 (2.59, 5.31)^***^	3.48 (2.42, 4.99)^***^
Age, years								
≤34	14	704,429	1.99	66	713,152	9.25	2.31 (1.26, 4.22)^***^	2.17 (1.18, 3.99)^*^
35–49	5	560,303	0.89	37	553,971	6.68	3.42 (1.55, 7.52)^**^	3.41 (1.54, 7.54)^**^
>50	19	502,128	3.78	36	470,819	7.65	2.21 (1.39, 3.53)^***^	1.89 (1.17, 3.04)^**^

*Rate^#^, Incidence rate per 10,000 person years; Crude HR, relative hazard ratio*;

*Adjusted HR^†^, adjusted hazard ratio after control for sex and comorbidities of CAD, hypertension, diabetes, hyperlipidemia, CVA, heart failure, COPD, CKD, alcohol-related diseases, cirrhosis, and biliary stone*.

*CAD, coronary artery disease; CI, confidence interval; CKD, chronic kidney disease; COPD, chronic obstructive pulmonary disease; CVA, cerebrovascular disease; PY, person years; SD, standard deviation*.

* *P < 0.05*,

** *P < 0.01*,

*** *P < 0.001*.

**Figure 1:**
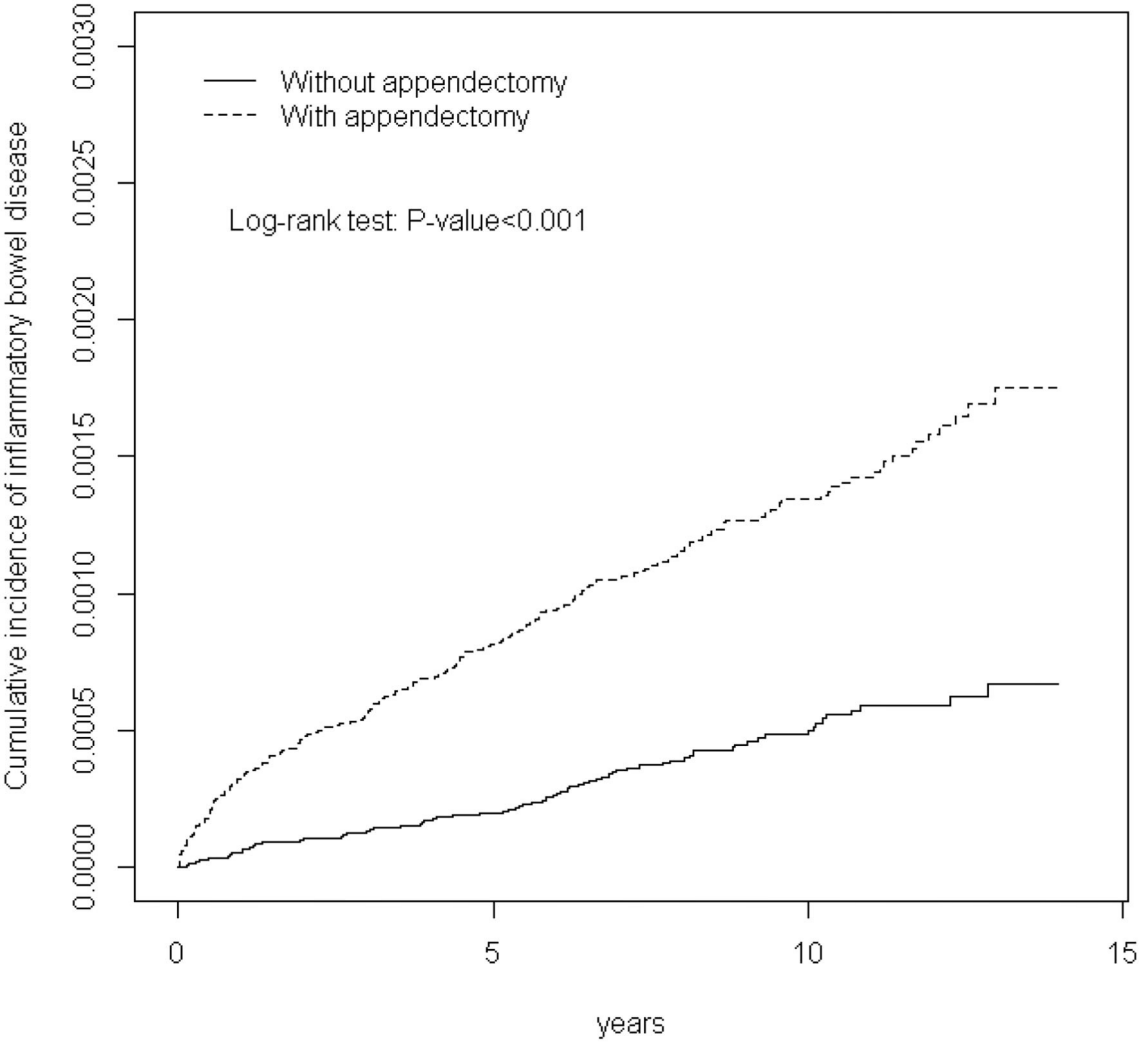
Cummulative incidence of inflammatory bowel disease in patients with appendectomy and comparison patients.

Figure 1 shows that the cumulative incidence of IBDs in patients with appendectomy was significantly higher than that of the comparison group (*P*-value < 0.001).

## Discussion

The nationwide cohort study indicated that the appendectomy cohort exhibited a higher incidence rate of IBDs than did the comparison cohort regardless of age, sex, and comorbidity. The appendectomy cohort had a 2.78-fold higher adjusted HR of IBDs (2.23-fold higher adjusted HR of UC and 3.48-fold higher adjusted HR of CD) than did the comparison cohort. Our results were comparable with a large case–control study, which showed a 1.6 and 2.5 times higher risk of UC and CD after appendectomy based on inpatient records from Veterans Affairs hospitals in the United States (15).

Andersson et al. (14, 16) reported that appendectomy is associated with an increased CD risk but a decreased risk of subsequent UC through an observational study of the Swedish Inpatient Registry. However, Andersson et al. indicated that only those patients who underwent appendectomy <20 years decreased a risk of UC development. Our study participants excluding patients <20 years may partially explained the controversial finding on the risk of UC. A retrospective case–control study from two Chinese hospitals did not show a significant negative association between appendectomy and UC occurrence (17).

Although IBD prevalence is higher in Western countries than in Taiwan, the incidence and prevalence of IBD have been rapidly increasing in Taiwan (10, 18, 19). The exact IBD pathogenesis remains to be elucidated, although IBD is generally considered to be related to genetic susceptibility and environmental factors (20, 21). Epigenetic modifications influenced by gut microbiota and diet may pay a role in IBD development (22, 23). Western-style diet may predispose people to IBD (24). Many people in Taiwan have shifted to a Western-style diet, which may be associated with an increased IBD incidence in Taiwan (25).

The vermiform appendix contains substantial lymphoid tissue and may act as a microbial reservoir for beneficial microbes to reinoculate the gut if required (6). The appendix provides a complex microbial environment for the homeostasis of immunologically and metabolically active organs (26, 27). Therefore, the appendix may serve as an organ to induce and maintain the mucosal immune system. An animal study indicated that appendectomy impairs intestinal immunity, which may be related to IBD development (28). Gut microbiota alteration in IBD may activate immune responses, interfere in homeostasis, cause tissue injury, decrease the mucus layer, and enhance microbial penetration and bacterial persistence in the gut tissue (29).

Anderson et al. conducted a cohort study by recruiting patients who underwent appendectomy from the inpatient registry database of the Swedish National Board of Health and Welfare and indicated that appendectomy due to appendicitis is associated with a decreased risk of subsequent UC (16). In contrast, no significant risk difference of UC was noted between appendectomy patients without inflammatory appendix and non-appendecomy controls (16). Frisch et al. suggested that patients aged <20 years at appendectomy for appendicitis or mesenteric lymphadenitis were related to reduced risk of UC development in Sweden and Denmark cohort studies (30). However, our study showed that the appendectomy cohort had increased UC risk regardless of whether the patient had appendicitis. Moreover, the UC risk was significantly higher in the appendectomy cohort than the non-appendectomy cohort irrespective of the age at appendectomy in the adulthood. The difference between Western studies and our study may be due to racial variances and dysregulated gut microbiota due to environmental insults (31).

The appendectomy cohort, irrespective of appendicitis, exhibited a considerably increased CD risk compared with the non-appendectomy cohort. The findings were consistent with those of previous studies (14). In addition, CD risk was significantly higher in the appendectomy cohort than in the non-appendectomy cohort irrespective of the age at appendectomy. The incidence rate of CD after appendectomy was the highest in the first 3 years. The risk of subsequent CD in the appendectomy cohort remained considerably higher than in the non-appendectomy cohort after 6 years following appendectomy.

This longitudinal cohort study estimated the incidence and risk of IBD in a large Asian population that underwent appendectomy. The study cohort could be followed throughout the follow-up period through NHIRD records because the NHI is mandatory and universal in Taiwan. However, several limitations should be noted when interpreting the results. First, coexistence of IBD and appendicitis was noted at appendectomy, which would be diagnosed by the pathologist. Second, the Western dietary habit of the study participants was not investigated in the current study. However, we controlled for the comorbidities of hyperlipidemia, diabetes, and hypertension to mediate the effect of a Western-style diet (32, 33). Third, familial history of IBD and smoking behavior in the study participants were not available in the NHIRD, which may have influenced the study results.

In summary, a nationwide cohort study indicated that the incidence and risk of CD and UC are higher in the appendectomy cohort than in the non-appendectomy cohort. The results highlight that clinicians must be aware that Asian patients undergoing appendectomy may develop CD or UC.

## Data Availability Statement

The original contributions presented in the study are included in the article/supplementary material, further inquiries can be directed to the corresponding author.

## Ethics Statement

The studies involving human participants were reviewed and approved by the Institutional Review Board of China Medical University Hospital (CMUH-104-REC2-115). The ethics committee waived the requirement of written informed consent for participation.

## Author Contributions

W-SC: conception and design. W-SC, SC, C-YH, and C-LL: administrative support, collection and assembly of data, data analysis and interpretation, and manuscript writing. All authors contributed to the article and approved the submitted version.

## Conflict of Interest

The authors declare that the research was conducted in the absence of any commercial or financial relationships that could be construed as a potential conflict of interest.

## References

[1] Tannoury J Abboud B. Treatment options of inflammatory appendiceal masses in adults. World J Gastroenterol. (2013) 19:3942–50. 10.3748/wjg.v19.i25.3942 23840138 PMC3703180

[2] Addiss DG Shaffer N Fowler BS Tauxe RV. The epidemiology of appendicitis and appendectomy in the united states. Am J Epidemiol. (1990) 132:910–25. 10.1093/oxfordjournals.aje.a115734 2239906

[3] Lin KB Chan CL Yang NP Lai RK Liu YH Zhu SZ. Epidemiology of appendicitis and appendectomy for the low-income population in Taiwan, 2003-2011. BMC Gastroenterol. (2015) 15:18. 10.1186/s12876-015-0242-1 25888516 PMC4329676

[4] Vitetta L Chen J Clarke S. The vermiform appendix: an immunological organ sustaining a microbiome inoculum. Clin Sci. (2019) 133:1–8. 10.1042/CS20180956 30606811

[5] Kooij IA Sahami S Meijer SL Buskens CJ Te Velde AA. The immunology of the vermiform appendix: a review of the literature. Clin Exp Immunol. (2016) 186:1–9. 10.1111/cei.12821 27271818 PMC5011360

[6] Randal Bollinger R Barbas AS Bush EL Lin SS Parker W. Biofilms in the large bowel suggest an apparent function of the human vermiform appendix. J Theor Biol. (2007) 249:826–31. 10.1016/j.jtbi.2007.08.032 17936308

[7] Im GY Modayil RJ Lin CT Geier SJ Katz DS Feuerman M. The appendix may protect against clostridium difficile recurrence. Clin Gastroenterol Hepatol. (2011) 9:1072–7. 10.1016/j.cgh.2011.06.006 21699818

[8] Kuenzig ME Benchimol EI Lee L Targownik LE Singh H Kaplan GG. The impact of inflammatory bowel disease in Canada 2018: direct costs and health services utilization. J Can Assoc Gastroenterol. (2019) 2(Suppl. 1):S17–33. 10.1093/jcag/gwy055 31294382 PMC6512251

[9] Kim YS Jung SA Lee KM Park SJ Kim TO Choi CH. Impact of inflammatory bowel disease on daily life: an online survey by the Korean association for the study of intestinal diseases. Intest Res. (2017) 15:338–44. 10.5217/ir.2017.15.3.338 28670230 PMC5478758

[10] Yen HH Weng MT Tung CC Wang YT Chang YT Chang CH. Epidemiological trend in inflammatory bowel disease in taiwan from 2001 to 2015: a nationwide populationbased study. Intest Res. (2019) 17:54–62. 10.5217/ir.2018.00096 30449079 PMC6361021

[11] Ramos GP Papadakis KA. Mechanisms of disease: inflammatory bowel diseases. Mayo Clin Proc. (2019) 94:155–65. 10.1016/j.mayocp.2018.09.013 30611442 PMC6386158

[12] Frisch M Johansen C Mellemkjaer L Engels EA Gridley G Biggar RJ. Appendectomy and subsequent risk of inflammatory bowel diseases. Surgery. (2001) 130:36–43. 10.1067/msy.2001.115362 11436010

[13] Gardenbroek TJ Eshuis EJ Ponsioen CI Ubbink DT D'Haens GR Bemelman WA. The effect of appendectomy on the course of ulcerative colitis: a systematic review. Colorectal Dis. (2012) 14:545–3. 10.1111/j.1463-1318.2011.02600.x 21689293

[14] Andersson RE Olaison G Tysk C Ekbom A. Appendectomy is followed by increased risk of crohn's disease. Gastroenterology. (2003) 124:40–6. 10.1053/gast.2003.50021 12512028

[15] Frisch M Gridley G. Appendectomy in adulthood and the risk of inflammatory bowel diseases. Scand J Gastroenterol. (2002) 37:1175–7. 10.1080/003655202760373380 12408522

[16] Andersson RE Olaison G Tysk C Ekbom A. Appendectomy and protection against ulcerative colitis. N Eng J Med. (2001) 344:808–14. 10.1056/NEJM200103153441104 11248156

[17] Chen D Ma J Luo S Lu L Wan X Ben Q. Effects of appendectomy on the onset and course of ulcerative colitis in Chinese patients. Gastroenterol Res Pract. (2018) 2018:2927891. 10.1155/2018/2927891 30524476 PMC6247428

[18] Ng SC Shi HY Hamidi N Underwood FE Tang W Benchimol EI. Worldwide incidence and prevalence of inflammatory bowel disease in the 21st century: a systematic review of population-based studies. Lancet. (2018) 390:2769–78. 10.1016/S0140-6736(17)32448-0 29050646

[19] Ng SC Bernstein CN Vatn MH Lakatos PL Loftus EV Jr Tysk C. Geographical variability and environmental risk factors in inflammatory bowel disease. Gut. (2013) 62:630–49. 10.1136/gutjnl-2012-303661 23335431

[20] Turpin W Goethel A Bedrani L Croitoru Mdcm K. Determinants of IBD heritability: genes, bugs, and more. Inflamm Bowel Dis. (2018) 24:1133–48. 10.1093/ibd/izy085 29701818 PMC6093195

[21] Liu JZ Anderson CA. Genetic studies of crohn's disease: past, present and future. Best Pract Res Clin Gastroenterol. (2014) 28:373–86. 10.1016/j.bpg.2014.04.009 24913378 PMC4075408

[22] Krautkramer KA Kreznar JH Romano KA Vivas EI Barrett-Wilt GA Rabaglia ME. Diet-microbiota interactions mediate global epigenetic programming in multiple host tissues. Mol Cell. (2016) 64:982–92. 10.1016/j.molcel.2016.10.025 27889451 PMC5227652

[23] Sartor RB Wu GD. Roles for intestinal bacteria, viruses, and fungi in pathogenesis of inflammatory bowel diseases and therapeutic approaches. Gastroenterology. (2017) 152:327–39.e4. 10.1053/j.gastro.2016.10.012 27769810 PMC5511756

[24] Castro F de Souza HSP. Dietary composition and effects in inflammatory bowel disease. Nutrients. (2019) 11:1398. 10.3390/nu11061398 31234325 PMC6628370

[25] Pan WH Wu HJ Yeh CJ Chuang SY Chang HY Yeh NH. Diet and health trends in taiwan: comparison of two nutrition and health surveys from 1993-1996 and 2005-2008. Asia Pacific J Clin Nutr. (2011) 20:238–50. 10.6133/apjcn.2011.20.2.14 21669593

[26] Guinane CM Tadrous A Fouhy F Ryan CA Dempsey EM Murphy B. Microbial composition of human appendices from patients following appendectomy. mBio. (2013) 4:e00366–12. 10.1128/mBio.00366-12 23322636 PMC3551545

[27] Kundu P Blacher E Elinav E Pettersson S. Our gut microbiome: the evolving inner self. Cell. (2017) 171:1481–93. 10.1016/j.cell.2017.11.024 29245010

[28] Dasso JF Howell MD. Neonatal appendectomy impairs mucosal immunity in rabbits. Cell Immunol. (1997) 182:29–37. 10.1006/cimm.1997.1216 9427807

[29] Zuo T Ng SC. The gut microbiota in the pathogenesis and therapeutics of inflammatory bowel disease. Front. Microbiol. (2018) 9:2247. 10.3389/fmicb.2018.02247 30319571 PMC6167487

[30] Frisch M Pedersen BV Andersson RE. Appendicitis, mesenteric lymphadenitis, and subsequent risk of ulcerative colitis: cohort studies in Sweden and Denmark. BMJ. (2009) 338:b716. 10.1136/bmj.b716 19273506 PMC2659291

[31] Abegunde AT Muhammad BH Bhatti O Ali T. Environmental risk factors for inflammatory bowel diseases: evidence based literature review. World J Gastroenterol. (2016) 22:6296–317. 10.3748/wjg.v22.i27.6296 27468219 PMC4945988

[32] Beigrezaei S Ghiasvand R Feizi A Iraj B. Relationship between dietary patterns and incidence of type 2 diabetes. Int J Prev Med. (2019) 10:122. 10.4103/ijpvm.IJPVM_206_17 31367285 PMC6639850

[33] Wang D He Y Li Y Luan D Yang X Zhai F. Dietary patterns and hypertension among chinese adults: a nationally representative cross-sectional study. BMC Public Health. (2011) 11:925. 10.1186/1471-2458-11-925 22168909 PMC3299712

